# The emerging intertwined activities of metabolism and epigenetics unveils culprits and prospects in cancer

**DOI:** 10.1038/s12276-025-01537-7

**Published:** 2025-09-12

**Authors:** Ayushi Verma, Anders M. Lindroth

**Affiliations:** Department of Cancer Biomedical Science, National Cancer Center, Graduate School of Cancer Science and Policy, Goyangsi, Republic of Korea

**Keywords:** Cancer metabolism, Cancer metabolism

## Abstract

The vast majority of cellular processes are interconnected in a manner that facilitates the overall function of the cell within its tissue environment. It has become evident that the dynamic interplay between metabolic processes and the regulation of genomic activities, including gene expression, is contingent on metabolite levels and factors that govern cellular distribution and compartmentalization. The advent of rapid technological and biophysical advances over the past two decades has yielded a compendium of factors, including metabolites and genes, that have provided extensive insight into their interrelationship. Here we discuss and summarize the many metabolites that have been experimentally shown to directly influence chromatin factors and epigenetic patterns. We aim to provide a comprehensive overview of the temporal and spatial dynamics of these processes within the cell, emphasizing the significance of metabolite abundance and the intricate orchestration of these processes during ontogeny and disease progression. The influence of lifestyle factors, such as diet and environmental exposures, on metabolite levels and their potential implications for therapeutic interventions is a subject of particular interest. The intricate interplay between metabolism and the epigenome in cancer offers a fertile ground for further research. By elucidating the manner in which metabolic fluctuations influence the epigenetic landscape, novel therapeutic approaches that target both metabolic and epigenetic pathways may emerge as promising avenues for cancer treatment.

## Metabolites shape epigenetic landscapes in cellular processes

Metabolism and epigenetics, once thought separate, are now seen as closely linked, especially in cancer. This Review explores how certain molecules, called metabolites, affect gene regulation in cancer. Researchers found that metabolites such as *S*-adenosylmethionine and acetyl-CoA play key roles in modifying DNA and proteins, influencing cancer growth. They used various methods to study these interactions, highlighting the importance of understanding how metabolism affects gene expression. The study shows that cancer cells often change their metabolism to support rapid growth, affecting the epigenome. For example, high levels of *S*-adenosylmethionine can lead to increased DNA methylation, which can silence tumor-suppressor genes. The research also discusses how diet and environmental factors can influence these metabolic pathways. The findings suggest that targeting these metabolic changes could offer new ways to treat cancer.

This summary was initially drafted using artificial intelligence, then revised and fact-checked by the author.

## Introduction

Metabolism and epigenetics, once seen as distinct processes, are now recognized as deeply intertwined, especially in cancer biology. Key metabolites such as *S*-adenosylmethionine (SAM), acetyl-CoA and nicotinamide adenine dinucleotide (NAD) serve as direct coenzymes or substrates for epigenetic enzymes, tightly linking cellular metabolic states to epigenetic regulation. Metabolic reprogramming in cancer often disrupts this balance, producing oncometabolites such as 2-hydroxyglutarate (2-HG), succinate and fumarate, which competitively inhibit epigenetic regulators and cause widespread epigenetic deregulation^1^. Beyond well-characterized oncometabolites, unconventional metabolites such as sarcosine, glycine, hypotaurine, kynurenine and methylglyoxal (MGO) are now being explored for their roles in epigenetic regulation and cancer progression, opening novel avenues for investigation^2–7^. Cellular metabolism transitions from coordinated processes during development to instability with aging, external exposures and genetic mutations. This metabolic disruption leads to epigenetic changes, clonal selection and disease. In cancer, metabolic adaptation is exploited for uncontrolled growth, with tumor cells hijacking developmental pathways and evading constraints through epigenetic plasticity^8^. Lifestyle factors such as diet, stress and environmental exposures influence cellular pools of key metabolites such as SAM, acetyl-CoA and NAD, further modulating the epigenome and providing additional opportunities for therapeutic intervention^9^.

Despite significant progress, studying transient metabolite–enzyme interactions remains challenging, but advanced techniques such as high-resolution structural studies and computational modeling are crucial for understanding these dynamics. This Review explores the interplay between metabolism and the epigenome in cancer, focusing on key metabolites, oncometabolites and dietary interventions. It addresses the challenges in studying metabolite–epigenome interactions and highlights emerging therapeutic strategies, aiming to provide insights into how metabolic shifts shape the epigenetic landscape and open new therapeutic avenues.

## Metabolites: the unsung authors of the epigenome

Epigenetic modifications, such as DNA methylation and histone modifications, serve as a dynamic ‘code’ that governs gene expression without altering the underlying DNA sequence^9^. Whereas enzymes such as DNA methyltransferases (DNMTs), histone acetyltransferases (HATs) and histone demethylases are the molecular ‘scribes’ of this code, metabolites act as both the ink and erasers, providing the necessary chemical groups for these modifications or removing them^10^. This interplay forms the foundation of how metabolites influences the epigenome.

## What are metabolites doing in the nucleus?

**Fig. 1:**
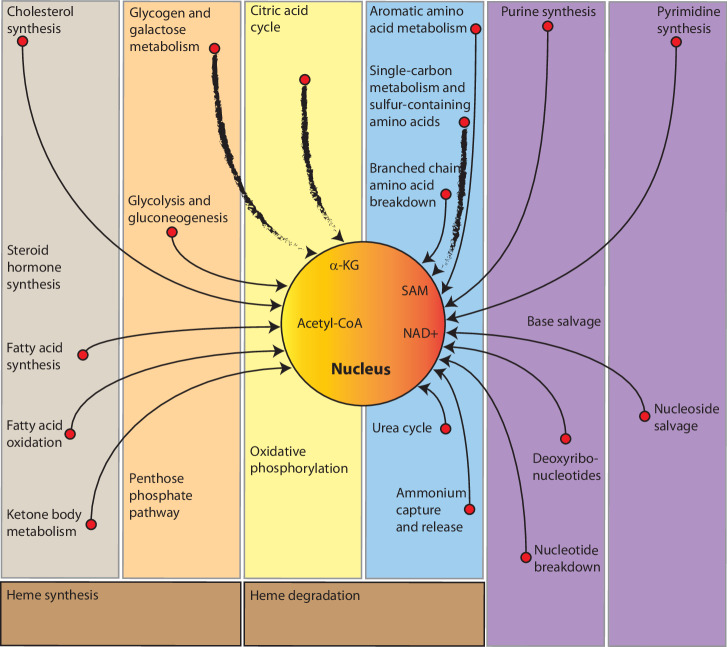
Summary of human metabolism pathways influencing epigenetic activities. All metabolic pathways are compartmentalized but share metabolites to neighboring pathways. The pathways that contribute to epigenetic activities are depicted with an arrows, where the thick-stroke lines contribute the most (Pathway of Human Metabolism, v10.23).

Metabolites, typically seen as intermediates in metabolic pathways, have a profound influence on epigenetic regulation (Fig. 1). They act as substrates, cofactors or inhibitors for key epigenetic enzymes, thereby directly shaping the epigenome^11^. Intriguingly, several metabolites bind directly to epigenetic modulators to regulate their activity, whereas others work indirectly by modulating enzyme availability or functionality. Beyond these roles, metabolites may also orchestrate the epigenome by coordinating the establishment and maintenance of epigenetic marks in response to changes in cellular metabolism, nutrient availability and environmental cues^12^.

## SAM: the master methyl donor in cancer epigenetics

**Fig. 2:**
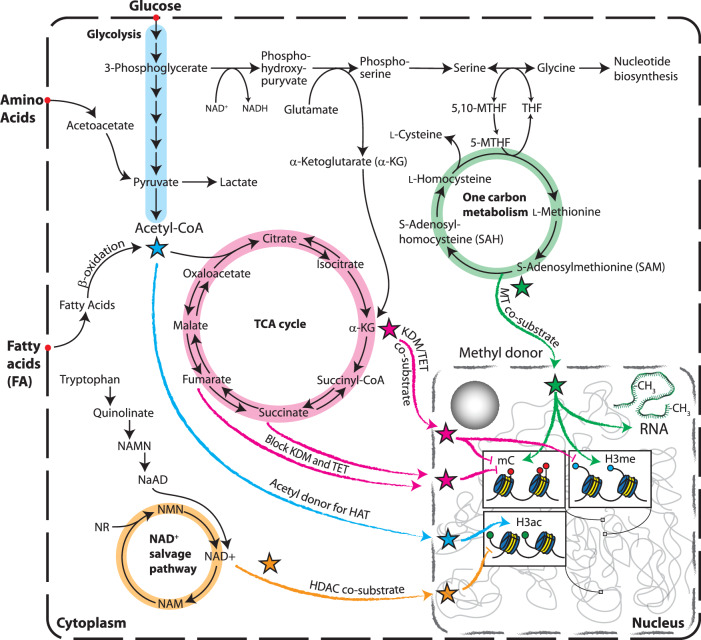
Illustration of the major metabolic pathways and their metabolites contributing to altered epigenetic activities. Several metabolites are cofactors or transient coenzymes (cosubstrates) to epigenetic enzymes, and several are inhibitors of their activities. The stars depicts major metabolic contributors in epigenetic regulation. Molecular abbreviations are NAMN Nicotinic acid mononucleotide, NaAD Nicotinic acid adenine dinucleotide, NMN Nicotinamide mononucleotide, NAD+ Nicotinamide adenine dinucleotide, NAM Nicotinamide, NR Nicotinamide riboside, MTHF Methylenetetrahydrofolate, THF Tetrahydrofolate, mC Methylated cytosine, H3me Methylated histone H3, H3ac Acetylated histone H3, HDAC Histone deacetylase, HAT Histone acetyltransferase, KDM Lysine demethylase, TET Methylcytosine dioxygenase, α-KG Alpha-ketoglutarate.

SAM is a central metabolite synthesized through the methionine cycle, serving as the universal methyl donor for DNA, RNA and histone methylation. SAM directly fuels the activity of DNMTs and histone methyltransferases (HMTs) and facilitates posttranslational modifications (PTMs) such as the methylation of lysine and arginine residues in nonhistone proteins (Fig. 2). SAM production is intricately linked to one-carbon metabolism, where nutrients such as methionine, serine and folate regulate its synthesis via methionine adenosyltransferase (MAT) enzymes. Once SAM donates a methyl group, it is converted into *S*-adenosylhomocysteine (SAH), a potent inhibitor of DNMTs and HMTs, highlighting the importance of maintaining the SAM/SAH ratio for proper epigenetic regulation^13,14^. High SAM levels enhance DNMT and HMT activity, promoting the methylation of DNA and histones, whereas the accumulation of SAH inhibits these enzymes. SAM availability also influences the activity of demethylases such as TETs and histone demethylases, which are sensitive to the metabolic environment^15^. Thus, SAM levels act as a key metabolic checkpoint controlling both methylation and demethylation processes. In cancer, the one-carbon metabolic pathway is frequently upregulated to support rapid cell division, with enzymes such as phosphoglycerate dehydrogenase (PHGDH) enhancing serine biosynthesis, which elevates SAM levels. This metabolic shift leads to the hypermethylation of tumor-suppressor genes and altered histone modifications, promoting tumor progression^16^. Key amino acid transporters, such as LAT1 and LAT4, are overexpressed in tumors to facilitate methionine uptake, essential for SAM production and subsequent cellular proliferation and differentiation^17^. In lung cancer, for example, LAT1 overexpression increases SAM abundance, which enhances the activity of HMT EZH2 and boosts H3K27me3 levels, further promoting tumor growth^18^.

DNA hypomethylation can activate oncogene transcription, thereby promoting carcinogenesis and tumor development. In cancers with low SAM, replenishment helps restore methylation balance rather than merely increasing methylation levels. SAM acts as a methyl donor in numerous methylation reactions and inhibits intracellular demethylase activity, which results in the hypermethylation of DNA. In a study on gastric cancer, SAM treatment was shown to induce hypermethylation of the vascular endothelial growth factor (*VEGF*)-C promoter. *VEGF-C* expression, typically elevated in gastric cancer, was downregulated following SAM treatment^19^. The *VEGF-C* promoters in MGC-803, BGC-823 and SGC-7901 gastric cancer cells, which normally express *VEGF-C*, were nearly unmethylated. However, after SAM treatment, these promoters were highly methylated, leading to the downregulation of *VEGF-C* expression. This resulted in a significant inhibition of cancer cell growth both in vitro and in vivo. SAM’s ability to regulate *VEGF-C* methylation underscores its potential as a therapeutic agent for silencing oncogenes and blocking cancer progression^19^. Dietary inputs and environmental factors influence SAM availability. For instance, restricting dietary methionine decreases SAM levels, suppresses EZH2 activity (an H3K27 methyltransferase) and inhibits tumor growth^20^. Similarly, reducing threonine metabolism lowers SAM synthesis, affecting H3K4me3 levels and cell proliferation^21^. The methionine salvage pathway, where methylthioadenosine is recycled into methionine and SAM, further supports SAM regeneration. However, the loss of methylthioadenosine phosphorylase in cancers impairs this process and sensitizes cells to PRMT5 inhibition, underscoring a therapeutic vulnerability^22^. SAM has demonstrated anticancer potential across various malignancies. In hepatocellular carcinoma (HCC), SAM selectively inhibits the growth, transformation and invasiveness of cancer cells, sparing normal liver cells. Methylomic and transcriptomic analyses reveal that SAM downregulates oncogenic pathways and hypermethylates metastasis-promoting genes such as *STMN1* and *TAF15*^10^. Similarly, in breast cancer and osteosarcoma, SAM reduces tumor proliferation, migration, invasion and metastasis in vitro and decreases tumor volume in vivo. Epigenome-wide association studies further reveal that SAM hypermethylates genes involved in prometastatic signaling and tumor progression, providing a mechanism for SAM’s therapeutic effects^23^. SAM’s potential in cancer therapy highlights its central role in linking metabolism to epigenetics, with implications for regulating key epigenetic modifications. Its synthesis and activity are influenced by intracellular metabolic pathways and external factors, such as nutrient availability, which directly modulate SAM levels. By targeting the one-carbon cycle or optimizing SAM production through dietary or pharmacological interventions, SAM can be harnessed for therapeutic purposes.

## Acetyl-CoA metabolism and epigenetic regulation in cancer

Acetyl-CoA is a central metabolite essential for processes such as histone acetylation, a key epigenetic regulator in cancer (Fig. 2). In cancer cells, fatty acid oxidation produces acetyl-CoA within mitochondria, which cannot directly cross the mitochondrial membrane. Instead, acetyl-CoA is converted to citrate in the tricarboxylic acid (TCA) cycle, exported to the cytosol and then converted back to acetyl-CoA by ATP citrate lyase (ACLY), linking fatty acid oxidation indirectly to ACLY activity and histone acetylation, independent of glucose metabolism^24^. In addition, the carnitine/acyl-carnitine shuttle system facilitates the transport of acetyl-CoA from mitochondria to the nucleus, where it supports epigenetic modifications and enhances metabolic flexibility^25^. In hypoxic tumor environments, acetate metabolism further boosts acetyl-CoA production through acetyl-CoA synthetase 2 (ACSS2), ensuring continuous histone acetylation even during metabolic stress^26^. Moreover, glutamine metabolism contributes to acetyl-CoA production through reductive carboxylation, particularly in cells with impaired oxidative phosphorylation^27^. In various cancers, elevated *ACLY* expression is linked to increased histone acetylation, driving oncogenes such as *MYC* and *HIF-1α*, which play pivotal roles in tumor progression^24^. Furthermore, acetyl-CoA metabolism also regulates immune evasion by promoting the expression of programmed cell death-ligand 1 (*PD-L1*), a key immune checkpoint protein. Studies have shown that high acetyl-CoA metabolism correlates with reduced antitumor immunity, whereas inhibiting ACLY reactivates cytotoxic T cells, enhancing the effectiveness of immunotherapy^28^. In pancreatic ductal adenocarcinoma, the acetyl-CoA metabolism is critical in tumor progression. A recent study observed that histone H4 acetylation is elevated in pancreatic acinar cells harboring *Kras* mutations, even before the appearance of premalignant lesions. This upregulation of acetyl-CoA in KRAS-mutant acinar cells supports acinar-to-ductal metaplasia, a crucial step in tumorigenesis. Interestingly, the pancreas-specific loss of ACLY inhibits acinar-to-ductal metaplasia and suppresses tumor formation^29^. In pancreatic ductal adenocarcinoma cells, the activation of AKT–ACLY signaling promotes histone acetylation, stimulating cell proliferation and tumor growth. Both BET inhibition and statin treatment, which target acetyl-CoA-dependent processes, suppress these effects, highlighting the therapeutic potential of targeting the acetyl-CoA metabolism in cancer^29^.

Collectively, these findings underscore the vital role of acetyl-CoA metabolism in promoting cancer cell plasticity, tumorigenesis and immune evasion. Given its central role in both metabolic and epigenetic regulation, targeting acetyl-CoA metabolism presents a promising strategy for treating KRAS-driven cancers and overcoming therapeutic resistance.

## Epigenetic enzyme regulation via cosubstrates

The function of many enzymes, including chromatin remodelers, depends on coenzymes such as NAD^+^. Specifically, the term ‘cosubstrate’ is used when a coenzyme temporarily binds to an enzyme and leaves it in an altered state. NAD^+^ is an oxidizing agent that accepts a hydride ion and a proton (H^+^) to become NADH or a phosphate group to become NADP. A lack of critical cosubstrates leads to altered epigenetic states that are correlated with disease states, such as cancer.

## NAD^+^ as a cosubstrate in histone deacetylation

Histone deacetylation, a crucial mechanism in regulating chromatin structure and gene expression, is mediated by four classes of histone deacetylases (HDACs): classes I, II, III and IV^11^. While classes I, II and IV HDACs are zinc dependent, class III HDACs, known as sirtuins, utilize NAD^+^ as a cosubstrate. During the deacetylation process, NAD^+^ is cleaved into 2′-*O*-acetyl-ADP-ribose and nicotinamide (NAM), with NAM being recycled into NAD^+^ through the NAD^+^ salvage pathway, a crucial source of NAD^+^ in the cell^30^.

## NAD^+^ and cancer metabolism

In rapidly proliferating cancer cells, the upregulation of NAM phosphoribosyltransferase (NAMPT) ensures sufficient NAD^+^ to sustain metabolic demands and support sirtuin function. In the salvage pathway, NAMPT converts NAM into NAM mononucleotide (NMN), which is then converted into NAD^+^ by NMN adenylyltransferases (NMNATs) (Fig. 2). NAMPT, the rate-limiting enzyme in this pathway, is frequently upregulated in cancers, including colorectal tumors, highlighting its potential as a therapeutic target^30,31^. Elevated NAMPT activity is crucial for maintaining NAD^+^ levels in rapidly proliferating cancer cells, supporting metabolic reprogramming, such as enhanced glycolysis, a hallmark of cancer cells^32^. This metabolic shift involves the conversion of NAD^+^ to NADH, which reduces the NAD^+^/NADH ratio, thereby diminishing sirtuin activity. The resulting decrease in sirtuin function leads to histone hyperacetylation and altered gene expression patterns that promote tumorigenesis^30^. Nutrient availability significantly influences NAD^+^ metabolism. For example, calorie restriction increases the NAD^+^/NADH ratio and enhances sirtuin-mediated deacetylation, which has been associated with tumor suppression^32^. Conversely, high-fat diets reduce NAD^+^ levels and sirtuin activity, contributing to a metabolic environment that supports cancer progression. Furthermore, the three NAD^+^-producing NMNATs—NMNAT1, NMNAT2 and NMNAT3—are localized in distinct cellular compartments: NMNAT1 in the nucleus, NMNAT2 in the Golgi complex and NMNAT3 in the mitochondria^33^. This compartmentalization ensures the precise regulation of NAD^+^ levels within specific cellular regions, which may influence sirtuin activity and chromatin dynamics. Given NAD^+^’s central role in regulating histone deacetylation and its impact on metabolic reprogramming, targeting NAD^+^ metabolism and its regulatory enzymes, particularly NAMPT, offers promising therapeutic avenues for cancer treatment. Modulating NAD^+^ levels could shift the epigenetic landscape, either promoting or inhibiting tumorigenesis, depending on the cancer cell’s metabolic context^33^.

## Oncometabolites: when good molecules go bad

Oncometabolites are metabolites that, when accumulated due to mutations in metabolic enzymes, contribute to the initiation and progression of cancer. These metabolites, typically involved in normal cellular processes, can become ‘bad’ molecules in the context of cancer, as they disrupt cellular metabolism, epigenetic regulation and overall cell function^34^. Oncometabolites can reprogram the epigenome by directly or indirectly interfering with key epigenetic modulators, thereby altering gene expression and promoting oncogenesis.

## 2-HG

The metabolite 2-HG, a metabolite structurally resembling α-ketoglutarate (α-KG), plays a key role in cancer, particularly in tumors with isocitrate dehydrogenase (IDH1/2) mutations. 2-HG exists as two enantiomers: ᴅ-2-HG (*R*-enantiomer), produced by mutant IDH1/2 enzymes, and ʟ-2-HG (*S*-enantiomer), which accumulates under conditions such as hypoxia and acidity or through the activity of enzymes such as PHGDH, MDH1/2 and LDHA^34^. Mutations in IDH1/2 lead to the neomorphic conversion of α-KG into ᴅ-2-HG, resulting in its accumulation and inhibition of α-KG-dependent dioxygenases such as TET2 and Jumonji-C histone demethylases. This leads to widespread DNA hypermethylation and histone modifications, contributing to tumorigenesis. In clear cell renal cell carcinoma, the loss of ʟ-2-HG dehydrogenase (L2HGDH), the enzyme responsible for degrading ʟ-2-HG, results in its accumulation as an oncometabolite and promotes oncogenic phenotypes^34^. In IDH1/2-mutant cancers, including gliomas and acute myeloid leukemia (AML), the mutant enzymes specifically produce ᴅ-2-HG, not ʟ-2-HG^35^. By contrast, wild-type IDH1/2 enzymes catalyze the oxidative decarboxylation of isocitrate to α-KG and do not produce 2-HG under normal physiological conditions. Accumulated ᴅ-2-HG competitively inhibits α-KG-dependent enzymes, leading to epigenetic alterations such as increased H3K9me3 and H3K27me3, gene silencing and impaired differentiation^36^. Inhibiting mutant IDH enzymes with drugs such as ivosidenib (AG-120) and enasidenib (AG-221) in IDH1/2-mutant cancers restores α-KG levels, reverses 2-HG-induced epigenetic changes and promotes differentiation, highlighting 2-HG as both a diagnostic marker and therapeutic target^37^.

## Lactate

Lactate, a key metabolic byproduct of glycolysis, is critical in cancer metabolism. Glycolysis, the process by which cells generate energy in the absence of sufficient oxygen, a hallmark of cancer cells known as the Warburg effect, leads to lactate accumulation^38^. Lactate acidifies the tumor microenvironment, promoting invasion and metastasis, while also acting as a signaling molecule. It also induces histone lactylation, an epigenetic modification that alters chromatin structure and gene expression^38^. Lactate-driven histone lactylation regulates cancer progression by promoting oncogene activation. In breast cancer, it enhances *c-Myc* expression and drives oncogene splicing via SRSF10, fueling tumor cell proliferation^39^. Similarly, in AML, lactate accumulation promotes *PD-L1* expression via the STAT5–lactate–PD-L1 axis, facilitating immune evasion and resistance to immunotherapy^40^. In HCC, lactylation affects TCA cycle enzymes, linking metabolism and epigenetic regulation^41^. In ocular melanoma, increased histone lactylation correlates with poor prognosis, enhancing *YTHDF2* expression, destabilizing tumor-suppressor mRNAs and accelerating tumor growth^42^. These findings highlight lactate as a key oncometabolite in cancer, driving metabolic reprogramming and epigenetic changes that promote tumor progression. Targeting lactate-driven pathways offers promising therapeutic strategies to inhibit cancer growth.

## Succinate

Succinate dehydrogenase (SDH) is a key in mitochondrial metabolism and tumor suppression. Mutations in SDH subunits impair succinate oxidation, leading to succinate accumulation, which activates oncogenic pathways such as STAT3 and EMT in colorectal cancer and drives angiogenesis in gastric cancer through VEGF signaling via GPR91^43^. Succinate reprograms T cell metabolism and epigenetics to promote proinflammatory states. It also alters histone and DNA methylation by inhibiting α-KG-dependent dioxygenases, such as TET enzymes and histone demethylases^44^. Despite these findings, succinate’s epigenetic effects appear context dependent, as several studies observed limited DNA methylation changes in succinate-treated cells^45,46^. Moreover, high succinate levels in HepG2 cells increase caspase activity with minimal genotoxicity, whereas fumarate induces DNA fragmentation and chromosomal instability, highlighting distinct oncogenic mechanisms^45^. These findings underscore the role of succinate as a retrograde signaling molecule, that is, a mitochondria-derived metabolite that communicates mitochondrial dysfunction or metabolic status to the nucleus and cytoplasm. Succinate has been demonstrated to promote cancer progression and immune modulation via metabolic, epigenetic and oncogenic pathways through such signaling. This provides new therapeutic insights.

## Fumarate

Fumarate, a key TCA cycle intermediate, is crucial for cellular metabolism. Its accumulation due to fumarate hydratase (FH) deficiency or mutation drives oncogenesis, making fumarate a significant oncometabolite^47^. Fumarate is normally metabolized by FH. In the event that FH loss has been rendered inactive, there is an accumulation of fumarate, which disrupts metabolic balance and contributes to the progression of cancer. As an oncometabolite, fumarate stabilizes HIF-1α by inhibiting prolyl hydroxylases, promoting angiogenesis and tumor progression through genes such as *VEGF* and impacting epigenetic regulation, DNA repair and immune evasion^3,47,48^. Fumarate inhibits TET enzymes, impairing DNA demethylation and causing DNA hypermethylation, which silences tumor-suppressor genes and activates oncogenes, further promoting cancer progression^49^. Fumarate-driven epigenetic alterations promote a malignant phenotype, enhancing cell proliferation, apoptosis resistance and metastasis. It also impacts DNA repair by interacting with histone variant H2A.Z at DNA double-strand breaks, inhibiting KDM2B and enhancing DNA-PK accumulation, which promotes nonhomologous end-joining for DNA repair and cell survival^47,49^. Fumarate supports DNA repair, maintaining genomic stability and cancer cell survival, especially after radiation. It also influences the tumor microenvironment, modulating immune cell function and promoting immune evasion. Accumulation due to *FH* mutations or loss is linked to hereditary cancers such as hereditary leiomyomatosis and renal cell carcinoma, driving aggressive tumors with early metastasis and poor prognosis^47,50^. Fumarate is also implicated in sporadic cancers, such as clear cell carcinomas and colorectal cancer, where its accumulation drives DNA hypermethylation and epigenetic changes, contributing to the malignant phenotype^47,51^. In clear cell carcinoma, FH inactivation causes fumarate accumulation, altering metabolism, promoting angiogenesis and enhancing cancer cell survival, accelerating tumor progression. Fumarate’s dual role in epigenetics and DNA repair highlights its potential as a therapeutic target for tumor progression and therapy resistance^47,51^.

## Modifications induced by oncometabolites

Oncometabolites such as ʟ-lactate drive cancer progression by inducing various PTMs, including lactylation. One key example is the ʟ-lactylation of adenylate kinase 2, which reduces its activity, disrupting energy homeostasis and HCC cell proliferation^41^. In addition, ᴅ-lactylation, a ᴅ-lactate-related modification, involves the nonenzymatic transfer of ᴅ-lactate to lysine residues. It arises from MGO conversion to lactoylglutathione by glyoxalase 1 (GLO1), followed by hydrolysis by glyoxalase 2 (GLO2), producing ᴅ-lactate. ᴅ-lactylation predominantly affects glycolytic enzymes, leading to a global reduction in glycolytic activity, with nearly 350 proteins identified as modified^52^.

Beyond lactate, fumarate and succinate, key TCA cycle metabolites, contribute to additional PTMs such as succination and succinylation. In succination, fumarate forms adducts with cysteine thiol groups, such as in KEAP1, which stabilizes NRF2 and activates an antioxidant program, aiding tumor survival under oxidative stress^53^. Other important targets of succination include the tumor suppressor PTEN and gasdermin D, with implications for apoptosis and inflammatory cell death, respectively^54^. Furthermore, fumarate-induced succination depletes glutathione, disrupting the cellular redox balance and contributing to cancer progression^55^. Similarly, succinylation, the transfer of succinyl groups from succinyl-CoA to lysine residues, regulates chromatin dynamics and mitochondrial protein function, influencing key processes in cancer development^56^. Elevated succinyl-CoA, driven by *SDH* mutations, leads to aberrant succinylation, contributing to apoptosis resistance and metabolic reprogramming, especially in *IDH1*/*IDH2*-mutant cancer cells^56^. Collectively, oncometabolites such as lactate, fumarate and succinate contribute to PTMs (specifically lactylation, succination and succinylation, respectively) that link metabolic reprogramming with epigenetic changes. These modifications influence cellular behavior, promoting tumorigenesis by rewiring metabolic and regulatory networks. Their interplay underscores their role in cancer progression and as potential therapeutic targets. A summary of oncometabolites and their effects on cancer epigenetics is provided in Table 1.

**Table 1:** Key oncometabolites and their impact on cancer epigenetics.

Oncometabolite	Mutation in gene	Key roles	Associated cancers	Epigenetic effects	References
D-2-HG	IDH1/2	Mutant IDH enzymes overproduce 2-HG (1) Inhibits α-KG-dependent dioxygenases (2) Promotes DNA/histone hypermethylation	Gliomas, AML, clear cell renal cell carcinoma, RCC	DNA/histone hypermethylation (1) CpG island methylator phenotype in gliomas	^34,94,95^
ʟ- or D- (ʟ-2-HG)	L2HGDH	Mutant L2HGDH enzymes overproduce 2-HG (1) Inhibits α-KG-dependent dioxygenases (2) Promotes DNA/histone hypermethylation	Brain tumor, RCC	DNA/histone methylation	^34^
Lactate	MCT/LDH	Product of glycolysis (1) Alters tumor microenvironment (2) Histone lactylation	Breast cancer, AML, HCC, ocular melanoma, lung cancer, prostate cancer	Histone lactylation/dysfunction of histone acetylation alters gene expression (1) Activates oncogenes (2) Enhances immune evasion	^5,38,39,41,42,96^
Succinate	SDH mutations impair Krebs cycle (1) Succinate accumulation (2) Immune modulation	SDH	Colorectal cancer, gastric cancer, paraganglioma, RCC	DNA hypermethylation (1) Inhibits TET/histone demethylases (2) Alters H3K27me3	^43,45,46^
Fumarate	FH deficiency leads to fumarate buildup (1) Affects DNA repair/angiogenesis	FH	Hereditary leiomyomatosis and renal cell carcinoma, clear cell carcinoma, colorectal cancer	DNA hypermethylation (1) Histone modification (H3K36 dimethylation) (2) Silences tumor-suppressor genes	^47–51,54,55^

## Uncovering metabolic drivers beyond tradition

### Sarcosine

Sarcosine (*N*-methylglycine), a metabolite linked to one-carbon metabolism, has been explored as a potential biomarker for prostate cancer progression. Initial studies showed that urinary sarcosine levels increased with cancer progression and metastasis, raising its diagnostic potential. However, later research revealed inconsistencies, as sarcosine failed to reliably differentiate prostate cancer from benign conditions or healthy individuals, offering no advantage over existing markers such as PCA3^57–59^. Despite its limited diagnostic value, a large study found a modest link between high serum sarcosine levels and reduced prostate cancer risk, highlighting the complexity of its role in cancer biology^59^. Beyond being a potential biomarker, sarcosine has been shown to affect epigenetic regulation. It increases levels of SAM, a key methyl donor and promotes CpG island methylation in prostate cells^60^. These findings suggest that sarcosine may act as an epigenetic modifier, influencing gene silencing and cellular behavior in prostate cancer. Although its diagnostic value is limited, its potential role in cancer epigenetics warrants further research into its biological functions and therapeutic implications.

### Glycine

Untargeted liquid chromatography–tandem mass spectrometry metabolomics of NCI-60 cancer cell lines highlights glycine’s crucial role in cancer cell proliferation^6^. Glycine supports purine biosynthesis and provides one-carbon units for DNA and histone methylation, essential for epigenetic regulation, by contributing to SAM synthesis^6^. Cancer cells amplify glycine dependence by upregulating transporters for glycine, serine, methionine and glutamine, driving metabolic and epigenetic reprogramming^6^. In non-small-cell lung cancer, glycine decarboxylase (GLDC) overexpression rewires glycolysis and glycine metabolism, driving tumorigenesis through metabolic and epigenetic shifts. Sarcosine supplementation restores proliferation in GLDC-deficient cells, highlighting cancer’s metabolic adaptability^61^. These finding positions glycine as a potential oncometabolite, bridging metabolism and epigenetic regulation in cancer. However, its role in tumorigenesis remains unclear, requiring further research to uncover its mechanisms and therapeutic potential.

### Hypotaurine

Hypotaurine emerges as a key oncometabolite in glioblastoma (GBM), with levels increasing alongside tumor grade, as revealed by capillary electrophoresis–mass spectrometry. Elevated homocysteic acid, which inhibits cysteine sulfinic acid decarboxylase, disrupts the conversion of cysteinesulfinate to hypotaurine and results in U-251 GBM cell arrest, highlighting the metabolic dysregulation in GBM progression^4^.

Hypotaurine drives GBM progression by inhibiting HIF-1α hydroxylation, activating hypoxia signaling and promoting a highly aggressive metabolic state. Its antagonist, taurine, counteracts this effect, suppressing hypotaurine synthesis and slowing tumor growth in xenograft models. In addition, 2-aminoethanethiol dioxygenase (ADO) catalyzes hypotaurine production from cysteamine, with its expression tightly linked to GBM progression. CRISPR–Cas9 knockout of *ADO* significantly impairs GBM cell proliferation and tumor growth, highlighting hypotaurine as a critical oncometabolite and potential therapeutic target^13,62^. These findings establish hypotaurine as a potential diagnostic and prognostic biomarker while positioning ADO and hypotaurine metabolism as promising therapeutic targets for GBM. Though its role in GBM progression is evident, its impact on epigenetic regulation remains unexplored. Given its influence on hypoxia signaling and metabolism, hypotaurine may also drive epigenetic changes affecting gene expression. Investigating its role in DNA methylation, histone modifications and chromatin remodeling could unveil new therapeutic avenues in epigenetic-based cancer treatments.

### Kynurenine

Kynurenine, a tryptophan-derived metabolite, is pivotal in cancer metabolism. It serves as a precursor for nicotinic acid and NAD synthesis, essential for energy metabolism and DNA repair^2^. Recent studies show that the oncogenic transcription factor MYC boosts the expression of tryptophan transporters (SLC1A5 and SLC7A5) and AFMID, promoting kynurenine synthesis and enhancing its metabolic flux in cancer cells^63^. Moreover, kynurenine activates the AHR transcription factor, which regulates growth-promoting genes, further linking it to cancer progression^2^. Elevated levels of kynurenine increase the synthesis of nicotinic acid, leading to higher concentrations of NADH and NADPH, which have been shown to be overexpressed in tumors harboring mutant p53^64^. Given its critical role in energy metabolism and DNA repair, kynurenine emerges as a potential oncometabolite. The enhanced cellular uptake of tryptophan and its subsequent metabolism through the kynurenine pathway has been confirmed using high-performance liquid chromatography–tandem mass spectrometry metabolomics, highlighting its relevance in cancer cell metabolism^63^. Kynurenine’s impact on NAD^+^ biosynthesis suggests it may influence epigenetic processes by affecting sirtuin enzymes, which regulate histone deacetylation and chromatin remodeling. This information suggests that kynurenine may be a promising target for therapeutic strategies that modulate both metabolic and epigenetic pathways in cancer.

### MGO

MGO, a byproduct of glycolysis, is elevated in cancer owing to increased glycolytic flux. As a precursor to advanced glycation end products (AGEs), MGO contributes to protein glycation, which is linked to cancer and other pathologies^65^. MGO modifies proteins at arginine residues, forming AGEs such as argpyrimidine and hydroimidazolone^66^. These modifications have been linked to cancer progression, particularly through the disruption of protein functions and cellular signaling pathways^65^. Recent studies reveal that MGO suppresses the detoxification enzyme glyoxalase 1, leading to nuclear localization of the transcriptional coactivator Yes-associated protein (YAP) and inhibition of the Hippo tumor-suppressor pathway in breast cancer cells, promoting uncontrolled cell proliferation^67,68^. Furthermore, MGO levels oscillate during the cell cycle, with higher concentrations observed in the G1 phase, suggesting its involvement in cell cycle regulation^3^. In addition, MGO has been shown to activate checkpoint kinases Chk1 and Chk2 in an AGE-dependent manner, highlighting its role in DNA damage response pathways^3,69^.

MGO modifies histones through glycation, potentially altering chromatin structure and gene expression in breast cancer. AGEs from MGO may disrupt DNA repair and transcription, influencing the epigenetic landscape. This positions MGO as a potential oncometabolite, warranting further investigation into its role in cancer metabolism and epigenetics. A summary of putative oncometabolites is provided in Table 2.

**Table 2:** Putative oncometabolites defined by metabolomics.

Oncometabolite	Primary role	Key findings	Epigenetic impact	Therapeutic implications	References
Sarcosine	One-carbon metabolism	Associated with prostate cancer progression	Elevates SAM; promotes CpG island methylation in prostate cells	Limited diagnostic utility; potential as an epigenetic modifier	^57,58,60^
Glycine	Serine–glycine one-carbon pathway	Supports purine biosynthesis, DNA methylation and tumor growth; GLDC drives metabolic reprogramming	Provides one-carbon units for SAM synthesis; links metabolism to epigenetic regulation	Targeting GLDC may inhibit tumor initiation; promising in non-small-cell lung cancer	^6^
Hypotaurine	Cysteine metabolism	Elevated in GBM; promotes tumor grade progression; inhibits HIF-1α hydroxylation	Unexplored but may influence hypoxia-induced epigenetic changes	ADO inhibition reduces tumor growth	^4,13,62^
Kynurenine	Tryptophan metabolism	Enhances NAD^+^ synthesis, promotes AHR signaling, supports energy metabolism and DNA repair	Affects histone deacetylation via NAD^+^-dependent sirtuins; potential chromatin-remodeling effects	Targeting tryptophan metabolism and kynurenine pathways may modulate tumor metabolism	^2,63,64^
MGO	Glycolysis byproduct	Drives protein glycation; promotes cell cycle progression and YAP activation in breast cancer	Modifies histones, disrupting chromatin structure and DNA repair	Glyoxalase 1 inhibition; potential for targeting AGE-related pathways	^9,65,67–69^

### Unraveling transient metabolite–protein interactions: challenges, emerging technologies and unanswered questions

Metabolite–enzyme interactions play a key role in metabolism, epigenetic regulation and cancer progression. However, their transient and dynamic nature, driven by weak noncovalent forces, makes them difficult to study. Conventional methods such as mass spectrometry and metabolomics provide valuable snapshots but struggle to link metabolites with their protein interactors owing to dissociation or low sensitivity for low-abundance complexes^9^. Similarly, chromatin immunoprecipitation, optimized for studying DNA–protein or histone–protein complexes, cannot stabilize or detect interactions involving small metabolites such as acetyl-CoA or SAM^70^. The discovery of oncometabolites, such as 2-HG in *IDH*-mutant cancers and fumarate/succinate in SDH- and FH-deficient cancers, underscores the critical role of dysregulated metabolite–enzyme interactions in epigenetic reprogramming and tumorigenesis^34^. Despite this, many oncometabolites remain undiscovered, and their contributions to epigenetic regulation and cancer progression are probably underestimated owing to methodological limitations. Emerging technologies, such as proximity labeling techniques (for example, BioID and APEX) and cross-linking mass spectrometry (XL-MS), offer new ways to capture these transient interactions^71^. XL-MS, for example, has successfully mapped acetyl-CoA interactions with chromatin-modifying enzymes, illustrating how metabolite–protein interactions are integral to both metabolism and epigenetic regulation^72^. However, XL-MS faces challenges, such as nonspecific interactions and the need for high concentrations of cross-linkers, which can perturb native cellular conditions^72^. Despite these advances, significant questions remain. What novel metabolite–protein interactions are critical to chromatin regulation and cancer progression but remain undetectable with current tools? Can selective cross-linkers or engineered probes be developed to stabilize metabolite–enzyme interactions under physiological conditions? How can spatially compartmentalized metabolites in distinct cellular regions be studied in their native environments? By integrating chemical biology, structural proteomics and computational modeling, we can uncover new oncometabolites as potential cancer therapeutic targets. Addressing these challenges is crucial for advancing our understanding of cancer metabolism and epigenetics.

### New frontiers in research

Recent advances in research tools are opening new frontiers in understanding the dynamic interplay between metabolites and epigenetic regulation. Single-cell multiomics technologies, which combine genomic, transcriptomic, proteomic and metabolomic data at the single-cell level, enable researchers to capture cellular heterogeneity and map complex molecular networks in unprecedented detail^73^. These platforms offer powerful insights into the spatial and temporal variations of metabolite levels, facilitating the identification of key metabolic regulators that drive epigenetic modifications^73^. Alongside this, artificial-intelligence-driven molecular simulations are proving essential in predicting metabolite–protein interactions and modeling complex biochemical pathways^74^. These tools may provide a computational framework to understand the mechanisms underpinning cellular metabolism and epigenetic changes with greater precision. Another major innovation is isotope-labeled metabolomics, which enables the tracking of specific metabolites in real time, providing insights into metabolic flux and its impact on gene expression and chromatin remodeling^75^. However, despite these advances, one of the most pressing challenges remains the integration of real-time metabolite tracking with epigenomic profiling. To truly capture the dynamic relationship between metabolism and epigenetics, future research must develop approaches that can synchronize metabolic changes with chromatin modifications as they occur in living cells. Integrating these data in a seamless and real-time manner would provide deeper insights into how metabolic reprogramming shapes gene expression, cellular fate and disease progression, particularly in cancer and other metabolic disorders.

### Order to chaos as revealed by metabolic shifts in aging and cancer

**Fig. 3:**
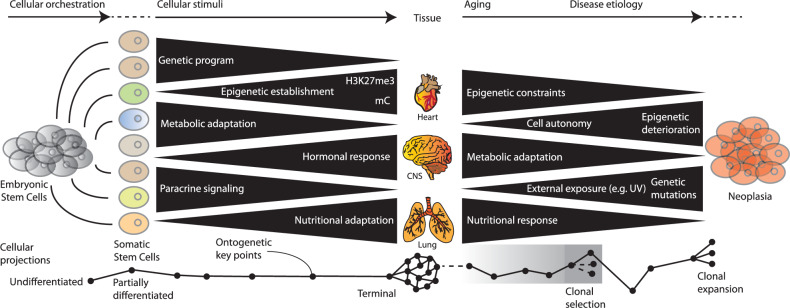
The cellular continuum of ontogeny and disease etiology. Broad categories of events and their quantitative contribution over time from embryonic stem cells to adult stem cells to functional tissue. The homeostasis and integrity of tissues in the body, in large part driven by age-related processes, contributes to disease etiology. Cancer develops when homeostasis is compromised and chaos ensues, leading to processes that resemble the events that allow embryonic stem cells to proliferate, awaiting signaling leading to cellular orchestration and differentiation.

Cellular metabolism undergoes dynamic transitions throughout development, from embryonic stem cells to differentiated tissues^8^. During early development, metabolic and epigenetic programs are highly coordinated, ensuring controlled differentiation and tissue homeostasis^8^. This structured process is maintained by precise genetic programs, metabolic adaptation and paracrine signaling, allowing cells to respond to physiological stimuli in an orderly manner. However, as tissues age, external exposures (such as ultraviolet (UV) radiation), hormonal fluctuations and accumulated genetic mutations introduce metabolic instability. These disruptions contribute to epigenetic deterioration, clonal selection and eventually disease etiology^76^ (Fig. 3). Cancer represents an extreme example of this shift, where metabolic adaptation is no longer regulated but exploited for uncontrolled growth. Tumor cells hijack developmental metabolic pathways; however, instead of following an orchestrated differentiation program, they remain in a state of epigenetic plasticity, evading cellular constraints. Understanding how these metabolic networks transition from order to chaos, particularly in aging and neoplasia, could provide critical insights into therapeutic strategies aimed at restoring homeostasis and targeting metabolic vulnerabilities in cancer progression (Fig. 3).

### Insights into DNMT–metabolite interactions and lipid metabolism

As discussed, distinct metabolites directly influence epigenetic modifications, mostly as cofactors. However, metabolites could also influence the epigenome as competitive and/or noncompetitive inhibitors of chromatin remodelers, possibly through altered metabolic output and shift in concentration leading to unwanted protein–metabolite interactions. We performed an untargeted metabolite analysis of cancer cell lines overexpressing FLAG-tagged DNMT3A and DNMT1, followed by co-immunoprecipitation, which provided us with chemical categories of molecules that would follow this pattern. Preliminary analysis based on UV and dimethyl pimelimidate cross-linking revealed associations with lipid-like molecules and organic metabolites using mass spectrometry. These interactions suggest that abundant metabolites could act as reversible inhibitors that negatively influence DNMT activity. Lipid-like molecules may influence enzymatic function as competitive inhibitors through hydrophobic or electrostatic interactions, potentially altering DNA methylation dynamics. Organic molecules, including aromatic or amino acid derivatives, could act as both competitive inhibitors or allosteric regulators, interfering with substrate binding or catalysis. Supporting this, a recent epigenome-wide association study of leukocyte DNA methylation identified significant associations with metabolic measures, particularly those related to lipoproteins. The study highlighted CpG sites in genes such as *ABCG1* and *PHGDH*, which were linked to lipid metabolism and metabolic diseases, including obesity and myocardial infarction. These genes also play a direct role in lipid transport (ABCG1) and amino acid metabolism (PHGDH), thereby highlighting a regulatory feedback loop. This finding lends further support to the notion that epigenetic regulation, facilitated by metabolites, is crucial for lipid metabolism and its associated disease pathways^77^. Furthermore, research examining the effects of high-glucose culture conditions on hepatocytes indicated that 25-hydroxycholesterol activates DNMT1, modulating genes involved in lipid metabolism. This suggests a direct role of metabolites, such as 25-hydroxycholesterol, in epigenetically regulating lipid metabolism. Similarly, another study found that maternal circulating lipids correlate with infant DNA methylation patterns, further supporting the hypothesis that metabolites have long-lasting epigenetic effects influencing metabolic health^78^. In addition, a study tracking 40 mother–infant dyads found significant correlations between maternal plasma metabolites, such as very-long-chain fatty acids and medium-chain acylcarnitines, and infant DNA methylation patterns^79^. These findings suggest maternal metabolites shape fetal epigenetics and long-term health. Exploring DNMT–lipid interactions through lipidomics and structural proteomics could reveal novel metabolic–epigenetic mechanisms, paving the way for targeted therapies in metabolic disorders and cancer.

### Dietary influence on epigenetics and cancer prevention

Dietary patterns influence cancer development and prevention by modulating epigenetic and metabolic pathways. Bioactive compounds from food interact with stress and environmental factors, shaping the epigenetic landscape. Cancer is marked by aberrant DNA methylation, characterized by global hypomethylation, which contributes to chromosomal instability and can lead to the activation of oncogenes. Simultaneously, gene-specific hypermethylation, particularly in the regulatory regions of tumor-suppressor genes, results in their silencing and promotes tumorigenesis^80^. This dual nature of DNA methylation underscores the potential of diet to counteract these changes, offering avenues for chemoprevention strategies^80^ Bioactive compounds such as epigallocatechin gallate (green tea), curcumin (turmeric) and quercetin (apples, onions) modulate epigenetics. Epigallocatechin gallate inhibits DNMTs, restoring tumor-suppressor genes such as *CDKN2A* and *MGMT*^81^. Similarly, quercetin and curcumin synergistically trigger global hypomethylation and apoptosis in cancer cells by disrupting mitochondrial function and cell cycle regulation^80,82,83^. Among dietary bioactives, phytoestrogens such as stilbenes, including resveratrol and pterostilbene and isoflavones such as genistein, act as natural endocrine modulators with significant epigenetic effects. The effect of stilbenes on the remodeling of DNA methylation and histone modifications have been demonstrated in the context of breast cancer and other models. These effects include the silencing of oncogenes and the restoration of tumor-suppressor activity^84–86^. Notably, despite growing evidence of their epigenetic effects, the influence of such compounds on epigenetic metabolites and their direct connection to chromatin remodeling remains insufficiently studied. However, challenges such as poor bioavailability and the need for effective systemic concentrations of these compounds limit their therapeutic application^80^. Advancing drug delivery and dietary formulations is key to overcoming these limitations. Environmental pollutants, tobacco smoke and endocrine disruptors further amplify epigenetic disruptions, worsening dietary imbalances^12^. Chronic stress disrupts epigenetic regulation by activating the hypothalamic–pituitary–adrenal axis, elevating cortisol and altering DNA methylation and histone modifications^87^. These stress-induced epigenetic changes can synergize with environmental carcinogens to drive tumorigenesis, underscoring the need to address psychosocial and environmental factors alongside diet. For example, one-carbon metabolism, crucial for DNA, RNA and histone methylation, relies on B vitamins (that is, B_6_, B_9_ and B_12_), methionine and choline^80,83^. Nutrient deficiencies and genetic polymorphisms, such as the *MTHFR* C677T variant, represent a significant cause for concern. This prevalent mutation has been demonstrated to reduce the activity of the methylenetetrahydrofolate reductase enzyme, thereby impairing folate metabolism^83^. Elevated homocysteine levels, impaired DNA repair and weakened immune responses are among the downstream effects of such disruptions^10,44^. Environmental toxins, such as heavy metals and persistent organic pollutants, can also interfere with one-carbon metabolism, compounding these^12^. Optimizing methyl-donor intake through diet may restore epigenetic balance and reduce cancer risk. Nutritional epigenomics offers a promising, accessible prevention strategy, with early life interventions supporting lifelong epigenetic stability, especially in high-risk populations^80^. Integrating bioactive dietary compounds with lifestyle changes—such as stress management, regular exercise and reduced exposure to environmental carcinogens—could synergistically reverse malignant epigenetic patterns^12^. Advancing precision nutrition for cancer prevention and therapy requires understanding the molecular mechanisms of dietary–epigenetic interactions, overcoming bioavailability challenges and exploring the interplay between genetic, epigenetic and environmental factors. This will enable targeted, personalized dietary strategies to reduce cancer incidence and improve outcomes.

### Dietary interventions as modulators of cancer metabolism and epigenetics

Recent studies suggest that dietary interventions, such as the ketogenic diet, may help combat cancer by targeting metabolic pathways and epigenetic modifications. This high-fat, low-carbohydrate diet lowers glucose and insulin levels while increasing ketone bodies such as β-hydroxybutyrate, which can inhibit tumor growth in certain models^88,89^. The ketogenic diet’s effects vary by context, with several studies linking it to increased tumor growth. Recent research shows it promotes metastasis in mice via BACH1, which activates prometastatic genes such as hyaluronidase 1. Inhibiting BACH1 mitigates this effect, while ATF4 enhances BACH1’s activity, highlighting potential risks for patients with cancer^90^.

Of note, methionine restriction alters DNA and histone methylation, influencing tumor suppression and chemotherapy sensitivity (for example, 5-fluorouracil). While promising, it may have adverse effects in some rodent models, highlighting the need for cautious application^91^. Similarly, glutamine, an essential amino acid for cancer cell metabolism, has been found to influence epigenetic marks, such as histone methylation, suggesting that glutamine modulation could be a potential strategy to disrupt cancer progression. Choline, a vital methyl donor in one-carbon metabolism, plays a critical role in cancer epigenetics. Its depletion disrupts DNA methylation and histone modifications, which can drive oncogenic reprogramming. Similarly, dietary fructose exacerbates tumorigenesis by promoting lipogenesis and histone acetylation, activating cancer-associated pathways^92^. In parallel, the gut-microbial metabolism of choline alters host methylation, metabolism and even behavior, as demonstrated in engineered microbial systems. Together, these findings emphasize the interplay between diet, microbial metabolism and epigenetic regulation in cancer and metabolic health. Recent advances in machine learning enable personalized health interventions by classifying individuals on the basis of metabotypes and lifestyle traits. These data-driven approaches might support precision medicine and chronic disease prevention^93^. These findings collectively suggest that dietary interventions targeting metabolic and epigenetic changes show promise as cancer therapies. However, their complex, context-dependent effects require further research to optimize clinical application and develop personalized treatments.

## Conclusions and future directions

The intricate relationship between metabolism and epigenetics represents a transformative area in cancer research, providing new insights into how cellular metabolic states shape gene regulation and tumor progression. This Review underscores the diverse roles of key metabolites, oncometabolites and dietary factors in modulating the epigenome, highlighting their potential as biomarkers and therapeutic targets. Despite significant advancements, several unanswered questions and technical challenges remain. One of the most pressing issues is the transient nature of metabolite–enzyme interactions, which complicates their study under physiological conditions. Current technologies, though innovative, often lack the sensitivity or precision required to capture these fleeting associations. Advancing methods such as isotope-labeled metabolomics, single-cell multiomics and spatially resolved epigenomics will be crucial to overcoming these barriers. Moreover, identifying unconventional metabolites with potential epigenetic roles, such as sarcosine and hypotaurine, opens new research avenues. Expanding the scope of the investigation beyond well-characterized molecules such as SAM and acetyl-CoA to explore the broader metabolome will probably uncover novel regulatory mechanisms and therapeutic opportunities. Cellular metabolism undergoes coordinated transitions from embryonic stem cells to differentiated tissues to maintain homeostasis. As tissues age, external exposures, hormonal changes and genetic mutations disrupt metabolic stability, contributing to epigenetic deterioration and cancer. In cancer, tumor cells exploit developmental metabolic pathways, maintaining epigenetic plasticity. Understanding how these metabolic networks shift from order to chaos, especially in aging and cancer, can inform therapeutic strategies targeting metabolic vulnerabilities. Regarding translational research, dietary interventions represent an exciting frontier for modulating cancer metabolism and epigenetics. Precision nutrition strategies tailored to individual metabolic profiles could optimize therapeutic outcomes, particularly when combined with pharmacological agents targeting epigenetic regulators. However, the context-dependent effects of dietary components underscore the need for personalized approaches based on tumor type, genetic background and metabolic status. Looking forward, integrating artificial-intelligence-driven computational modeling or machine learning with experimental research will be a game-changer, enabling the prediction and validation of metabolite–protein interactions at unprecedented scales. These tools could also facilitate the development of small-molecule inhibitors or activators specifically targeting metabolite-driven epigenetic dysregulation. Understanding the metabolism–epigenetics interplay can drive breakthroughs in cancer biology and personalized therapies. Bridging knowledge gaps and leveraging emerging technologies will unlock the full potential of this axis for cancer diagnostics, prevention and treatment.
